# Translation, cultural adaptation and pilot testing of a questionnaire measuring the factors affecting the acceptance of telemedicine by Greek cancer patients

**DOI:** 10.1371/journal.pone.0278758

**Published:** 2023-02-02

**Authors:** Nikolaos Papachristou, Ravikalis Vasileios, Pavlos Sarafis, Panagiotis Bamidis

**Affiliations:** 1 Medical Physics and Digital Innovation Laboratory, School of Medicine, Aristotle University of Thessaloniki, Thessaloniki, Greece; 2 School of Social Sciences, Hellenic Open University, Patras, Greece; 3 Psychiatric & Geriatric Clinic Galini, Kavala, Greece; 4 General Department of Lamia, University of Thessaly, Lamia, Greece; Mugla Sitki Kocman Universitesi, TURKEY

## Abstract

The emergency presented through the COVID-19 pandemic exposed the need to adopt remote, technology-driven solutions and make healthcare services more resilient. To do so, we need technological applications (i.e., telemedicine) that are designed and tailored to the end-users (i.e., chronic patients) needs and the type of healthcare service they get (i.e., cancer care). The requirements above are especially relevant to Greece, being a country with numerous sparsely populated regions (e.g., islands, regions at the borders) and a deteriorating access to healthcare for all citizens. Trying to address such diverse problems and needs, there have been multiple, different telemedicine and telecare projects in Greece in the past years. To support the future design and implementation of such endeavours, in this study we translated a questionnaire measuring the acceptance of telemedicine by patients and adapted it to the Greek context. We continued by running a small-scale pilot with 73 Greek women with breast cancer to assess the adapted instrument for its reliability and construct validity. The created questionnaire had good overall and internal reliability scores for most sub-scales. Factor analysis did not identify the same number of latent dimensions as the original theoretical model. Reverse wording items needing to be recoded were identified, and items that could be omitted in future versions of the questionnaire. Increasing the sample size for the purposes of a longitudinal study, the construct, convergent, and discriminant validity are elements to be further examined in future studies. It is envisaged that the creation of this questionnaire will support the adoption of telemedicine by Greek healthcare services into more routine areas of patient care provision.

## Introduction

The World Health Organization (WHO) has labeled COVID-19 a worldwide pandemic in March 2020 [1]. From the early days of the pandemic, it was made evident that governments would be obliged to concentrate on preventative techniques that decrease viral transmission because of the absence of specific therapies and vaccinations for the disease. The measures that have taken place, like social distancing, isolation, and quarantine, have provided a historically unparalleled challenge for healthcare organizations, which have been obliged to adapt their traditional modes of service delivery accordingly [2]. For example, these adjustments have resulted in medical visits, even treatments, being moved from hospitals to patients’ homes via the development of home care and the adoption of telemedicine [3], influencing both the urgent and ambulatory modes of healthcare services delivery accordingly [4]. The emergency presented caused an unprecedented need to identify and adopt efficiently remote, technology-driven solutions for supporting these modifications [5, 6]. Several papers, guidelines, and calls to action for healthcare professionals have been issued, during this period, on how to utilize telemedicine, telecare, and digital health interventions to treat and support all types of patients, COVID-19 included [7–11].

The aforementioned need to adopt telemedicine and telecare services has been quite prominent in oncology as COVID-19 has had a tremendous effect on cancer care services, patients, and professionals [12, 13]. According to an analysis by WHO, assessing the continuity of essential health services during the COVID-19 pandemic, there has been a worldwide disruption in cancer care (i.e., screening and treatment) in the last quarter of 2021, ranging from 5% to 50% depending on the country [13]. For example, patients with cancer have been reported to be more prone to negative outcomes after infection with the SARS-CoV-2 virus [14–16]. Screening programs and diagnostic services have been reduced or halted in many countries, causing the delay of many potential cancer diagnoses [17]. Finally, as some components of continuing treatment have been deprioritized and healthcare professionals have been repurposed to handle patients with COVID-19 [18]. cancer care services showed signs of dysfunction and oncology professionals signs of work-related stress [19, 20]. All of these issues led to the conclusion that the adoption of telemedicine-based interventions is an acceptable and reasonable alternative [21, 22].

It is noteworthy to mention here that telemedicine has been steadily developed and studied for more than two decades, prior to this sudden influx of interest. A recent systematic review from Bahlol Rahimi et al., 2018 [23] reported that telemedicine applications peaked between 1999 and 2017, while they were the most studied application area of information and communication technology (ICT) for end-user’s acceptance. Another recent systematic review by Lorenz Harst et al., 2019 [24] reported that perceived utility, social influence, and attitude were the factors that were most often associated with acceptance. Moreover, in the case of patients acceptance of telemedicine from their social surroundings was critical, as family and friends could encourage them to adopt telemedicine as a treatment option [24].

Regarding the geographical- and nation-wide interest for the adoption of telemedicine, several such telemedicine projects have been tested in the last two decades only in Greece [25–29]. Being a country with an intermediate level of integrated care maturity [30, 31], a large number of low population density areas (for example, islands and regions near the borders) [32] and access to healthcare services care that has become worse in the past decade due to economic reasons and COVID-19 [33–35], make Greece quite suitable for the implementation and wide adoption of telemedicine across its national healthcare system. Only in oncology, there have been several recent Greek studies examining: how mobile and smart-health solutions can enhance the quality of life of thyroid cancer patients [28], prostate and cancer patients [26]; how electronic Patient Reported Outcomes can support palliative cancer care [28] and symptom management during and after chemotherapy [26, 27].

Taking into account the special interest that telemedicine will always have for the Greek context both in the pre- and post-COVID era, as well as the above mentioned healthcare services’ gaps and suggestions for its adoption, we translated a questionnaire measuring the acceptance of telemedicine by patients and adapted it to the Greek context. We also ran a pilot to assess the adapted questionnaire’s reliability and get insights about its conformity to the original conceptual model. Considering the literature’s guidance that individual characteristics of the end users influence significantly their levels of telemedicine acceptance [24] as well as the disruption that COVID-19 caused to cancer care services, we ran the pilot with a homogeneous group of Greek breast cancer patients. As far as we know, this is the first such questionnaire and study for Greek patients.

## Materials and methods

This research was a cross-sectional, pilot study designed to translate into the Greek language and culturally adapt the original instrument created by Dehe Li et al., 2020 [36]. Being a pilot study, the adapted instrument was also verified for its reliability and assessed for its validity.

### Participants

The pilot’s population consisted of a convenience sample of Greek women with breast cancer. The data collection process was carried out in June 2021. An online, anonymous questionnaire was distributed with snowball sampling [37] through the association of women with breast cancer "Alma Zois", which is a Non-Governmental Organisation (NGO) of breast cancer patients based in the prefecture of Thessaloniki. The online questionnaire was distributed through online (i.e., emails, social media) and offline channels (i.e., word of mouth) of “Alma Zois”, and it was completed by 73 participants. All respondents completed the questionnaire in full.

### Original questionnaire

The questionnaire presented in this study was derived from the relevant scale developed by Dehe Li et al., 2020 [36]. The original instrument aimed to capture patients’ intention to use online health services based on the theory of planned behavior (TPB) [38], which is comprised of five domains: attitude toward the behavior, subjective norm, perceived behavioral control, behavioral intention, and actual behavior. According to TPB, people engage in certain activities according to their behavioral intention. To complement the subjective and social factors that may affect a patient’s behaviour in the healthcare settings (i.e., use of online services by a hospital), Dehe Li et al., 2020 [36] added sub-scales that were relevant to patient’s perceived severity of sickness, and perceived medical risk from using an online service by a hospital. The final instrument consisted of 35 items across 12 dimensions or sub-scales. These sub-scales were namely the following: perceived convenience (PC); perceived outcome (PO); perceived medical risk (PMR); perceived information risk (PIR); emotional preference (EP); perceived medical liability (PML); attitude toward the behavior (ATTB); subjective norm (SN); health consciousness (HN); perceived severity of disease (PSD); perceived behavioral control (PBC); behavioral intention (BI).

### Greek translation and cultural adaptation

The translation and cultural adaptation of the Greek questionnaire of this study were carried out following guides provided from the relevant literature [39–42]. More specifically, translation was accomplished via forward–backward transcription, the most generally used translation procedure for surveys and inventories [39]. One bilingual translator translated the 35 items of the original questionnaire. Another bilingual translator who did not know of the original instrument then back-translated the re-conciliated Greek version. Going forward in the process, two public health Greek bilingual experts compared the forward and backward translations with the original English version and applied appropriate modifications, enhancements, and cross-cultural adaptations, providing also comments and judgments about inaccuracies. An “intermediate version” of the questionnaire was completed based on the two Greek bilingual experts’ consensus on adaptations and comments.

The “intermediate version” of the translated and culturally adapted questionnaire was further refined through a cognitive debriefing process, which was used to identify any language problems and assess the degree of respondents’ understanding of the item’s content that was meant to be elicited [41]. Thus, a structured interview with 5 chronic patients was used to reveal inappropriately interpreted items and translation alternatives. The participants shared their thoughts on how well each item was explained, how relevant it was to their particular circumstance, how detailed the instructions were, and whether they could finish it on their own. They were also encouraged to make suggestions whenever necessary. With the feedback accumulated in this stage the project’s lead scientist amended and completed the final, Greek version of the questionnaire. You can find the template of this structured interview together with its English translation at S1 Table.

The final, translated and culturally adapted questionnaire, consisted of the same number of items and sub-scales, aiming to capture patient’s intentions to use an electronic health service provided by a hospital. You can find the Greek version of the questionnaire together with its English translation at S2 Table.

### Reverse worded items

Reverse worded items are phrased in a different direction from the ’normal’ items on the scale [43, 44]. For example, “normal” items can be phrased in a positive way according to the direction of the construct (i.e., “I am confident” on a scale about telemedicine). On the other hand, reverse worded items can be phrased in negative way, in the opposite way to the direction of the construct (i.e., “I am worried” on a scale about telemedicine).

Unfortunately, reverse wording may cause several problems in the completion of the items and the analysis of the results. For instance, respondents may be perplexed by reversed worded items as a result of the increased difficulty in reading the questions. [44] Also, reverse worded elements may introduce a method factor, leading in a scale that measures something other than what the researchers intended to assess [44].

For such reasons, these items need to be reverse coded (i.e., “completely agree” changes to “completely disagree”) before many types of analysis [43]. The correlation analysis of our questionnaire highlighted the existence of such reverse worded items. Wherever it was appropriate we reverse coded these item, providing this information at the results section of this report.

### Statistical analysis

All the analysis presented in this study were conducted using R [45]. For the respondents’ demographics, descriptive statistics were used. The questionnaire’s qualitative variables were expressed as relative percentages. Spearman coefficients were used to explore intercorrelations among the likert items of the subscales [46].

### Sampling adequacy and sphericity

Before exploring it, we tested if our sample was suitable for carrying out factor analysis. For this reason, we utilised the Kaiser-Meyer-Olkin (KMO) measure of sampling adequacy [47, 48] and Bartlett’s test of sphericity [47, 48]. For a KMO to be judged acceptable, it must be more than 0.5. Bartlett’s test of sphericity was done in order to identify the common factors and to determine whether or not the factor analysis model was adequate.

### Construct validity

Being a pilot study of a large questionnaire with 12 sub-scales (i.e., theoritical latent dimensions), our respondents’ sample size was too small for a confirmatory factor analysis (CFA) [49]. Instead, to assess the latent dimensions that might be hidden in the observed variables, exploratory factor analysis was used (EFA) [49, 50]. With these findings we made an initial qualitative assessment of how the data collected fitted the theoretical construct of the original questionnaire [36].

In relevance to the sample size needed for this analysis, it is noteworthy that there isn’t a consensus or a “gold standard” about the required sample size to conduct factor analysis. For example, some authors use a threshold based on the total sample size, while others base it on a ratio of the number of cases to the number of variables involved in the factor analysis [51, 52]. Factor analysis for this small sample, pilot testing study is based on the “rule of thumb” suggested by Kline. 1994 [52], where two subjects per variable is a satisfactory condition for our case.

Regarding the correct numbers of factors to retain, there is a plethora of statistical strategies available for dealing with this problem, and the outcomes of these methods may often be significantly different [53, 54]. Unfortunately, there is no single approach to be reliable in all instances [53, 54]. Thus, we employed multiple methods with the “Method Agreement procedure” and we selected the solution that had the highest consensus among them [48, 55]. We, also, present the scree plot of the Principal Component Analysis (PCA) eigenvalues extracted from all the 35 items of the questionnaire.

When evaluating the developed questionnaire, we utilized EFA with a weighted least squares (WLS) procedure and a varimax rotation [50], while its cumulative total of variance and factor loadings were used to appraise the construct validity. If the cumulative total of variance of the principal components selected accounted for more than 70% of the total variance, it was determined that the composition of the principal components as exogenous variables was compatible with the constructs of the questionnaire. If each item had a factor loading value of 0.50 or higher on one of the principle components but factor loading values of less than 0.50 on the other principal components, the validity of the planned questionnaire was deemed satisfactory, and no items were considered for future deletion.

### Reliability analysis

While Cronbach’s alpha is one of the most often used reliability metrics, its application has received significant criticism [56, 57]. As it is reported in numerous different studies, Cronbach’s alpha statistical assumptions (i.e., tau equivalence, continuous items with normal distributions, uncorrelated errors, uni-dimensionality) are not in sync with today’s statistical knowledge and practice [56, 57]. As a result, a variety of other metrics of reliability have been suggested. In our study, in order to assess the internal consistency and the reliability of the created scale, as well as the composite sub-domains with more than two items, we report Cronbach’s alpha together with Guttman’s Lambda 6 and McDonald’s ω. Finally, to assess the reliability of the two-item sub-domains in our questionnaire, we report the Spearman-Brown reliability [58]. For all these reliability metrics, taking into account that this was a pilot testing study of a newly translated and culturally adapted questionnaire, we used the “rule of thumb” cut points of ≥ .9 –Excellent, ≥ .8 –Good, ≥ .7 –Acceptable, ≥ .6 –Moderate, ≥ .5 –Poor.

### Ethics

The study got ethics approval from the Bioethics Committee of the Aristotle University of Thessaloniki, Greece.

## Results

### Sample characteristics

Because of the data collection method used (i.e., snowball sampling) we can not make an estimate of the response rate of the validation phase. In total 73 questionnaires were returned, all of them, fully completed. Among these 73 female cancer patients with breast cancer, the majority were aged between 50 and 59 (37%) or between 40 and 49 years (31.5%). As far as educational level is concerned, the majority was university graduates (69.9% in total; 50.7% had a bachelor degree), while 13.7% had a MSc degree and 4% a PhD. Concerning living alone or with others, the majority was living with others (80.8%). As regards monthly average income, 56.2% of participants had a higher average income than the median equivalised net income of Greece (8,781€ per year; 731,75€ per month) [59]. Finally, in relation to prior experience or use of telehealth services in the past, 43.8% of the participants answered yes (Table 1).

**Table 1:** Demographic characteristics of the participants (N = 73).

Characteristics	Value, n (%)
**Age (years)**	
19–29	1 (1.4)
30–39	9 (12.3)
40–49	23 (31.5)
50–59	27 (37)
≥60	13 (17.8)
**Education level**	
Up to secondary school	14 (19.2)
Post secondary Education	8 (10.9)
Bachelor	37 (50.7)
MSc	10 (13.7)
PhD	4 (5.5)
**Marital Status**	
Unmarried	15 (20.6)
Married	44 (60.3)
Divorced	12 (16.4)
Widow	2 (2.7)
**Living alone or with others**	
Living alone	14 (19.2)
Not living alone	59 (80.8)
**Monthly average net income, € ($ US)**	
<500 (550.61)	7 (9.6)
501 (551.71)– 1000 (1101.22)	25 (34.2)
1001 (1102.33) - 1500 (1651.84)	24 (32.9)
>1501 (1652.94)	17 (23.3)
**Medical insurance status**	
Having medical insurance	71 (97.3)
Not having medical insurance	2 (2.7)
**Prior experience/use of telehealth services in the past**	
Yes	32 (43.8)
No	41 (56.2)

### Descriptive statistics, correlation analysis and reverse worded items

**Fig 1:**
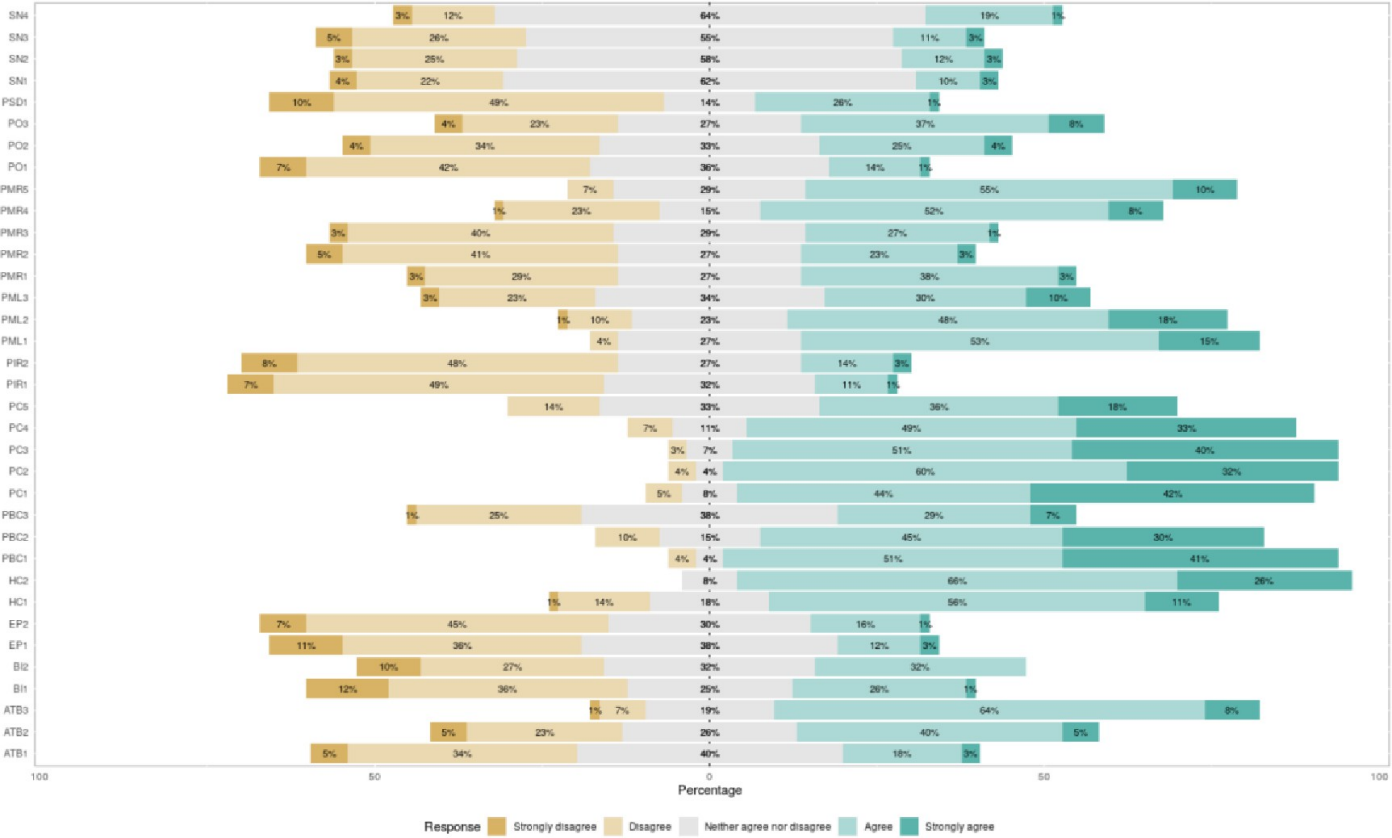
Summary of the questionnaire’s answers per item (i.e., subjective norm) and relevant questions (e.g., SN1, SN2, SN3, SN4). You can see the list of the questionnaire’s items in S1 Table. The 12 sub-scales of the questionnaire are: perceived convenience (PC); perceived outcome (PO); perceived medical risk (PMR); perceived information risk (PIR); emotional preference (EP); perceived medical liability (PML); attitude toward the behavior (ATTB); subjective norm (SN); health consciousness (HN); perceived severity of disease (PSD); perceived behavioral control (PBC); behavioral intention (BI).

**Fig 2:**
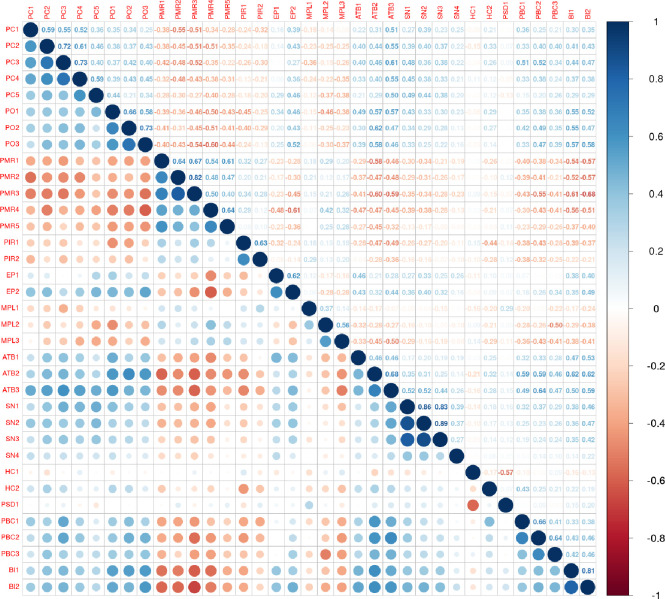
Spearman correlation analysis between all the ordinal, likert items of the translated and culturally adapted questionnaire. Each item is abbreviated in relation to the sub-scale it belongs. For example PC1, is the first item of the sub-scale perceived convenience (PC). You can see all the items, in full, at S1 Table. The 12 sub-scales of the questionnaire are: perceived convenience (PC); perceived outcome (PO); perceived medical risk (PMR); perceived information risk (PIR); emotional preference (EP); perceived medical liability (PML); attitude toward the behavior (ATTB); subjective norm (SN); health consciousness (HN); perceived severity of disease (PSD); perceived behavioral control (PBC); behavioral intention (BI).

There were no missing responses across the questionnaire from the 73 participants. In overall, the sub-scale of perceived convenience (PC) seemed to collect the most positive responses (or most high scores if these items were to be summed for each sub-scale) (Fig 1, S3 Table). Similarly, the sub-scales of health consciousness (HC), perceived behavioral control (PBC) and perceived medical liability had also a high relative percentage of positive responses (Fig 1, S3 Table). Correlation analysis (Fig 2, S1 Fig) showed that the five items (PMR1 to PMR5) in perceived medical risk (PMR), the two items (PIR1, PIR2) in perceived information risk (PIR), the three items (PML1 to PML3) in perceived medical liability (PML), the one item (PSD1) in perceived severity of disease (PSD), and the first item (HC1) in the health consciousness (HC) sub-scales had an opposite direction in relation to all the other items of the scale. Thus, these items were reverse coded for the rest of the analyses presented in this report.

### Sampling adequacy and sphericity

The KMO statistic test yielded a value of 0.77. This demonstrated that the sample size was likely sufficient for factor analysis [60, 61]. It was also found that the observed data was sufficiently spherical by Bartlett’s test, with a chi-square test result of 1842.91 (degree of freedom = 595, p < 0.001) suggesting that factor analysis was appropriate for the data.

### Construct validity

**Fig 3:**
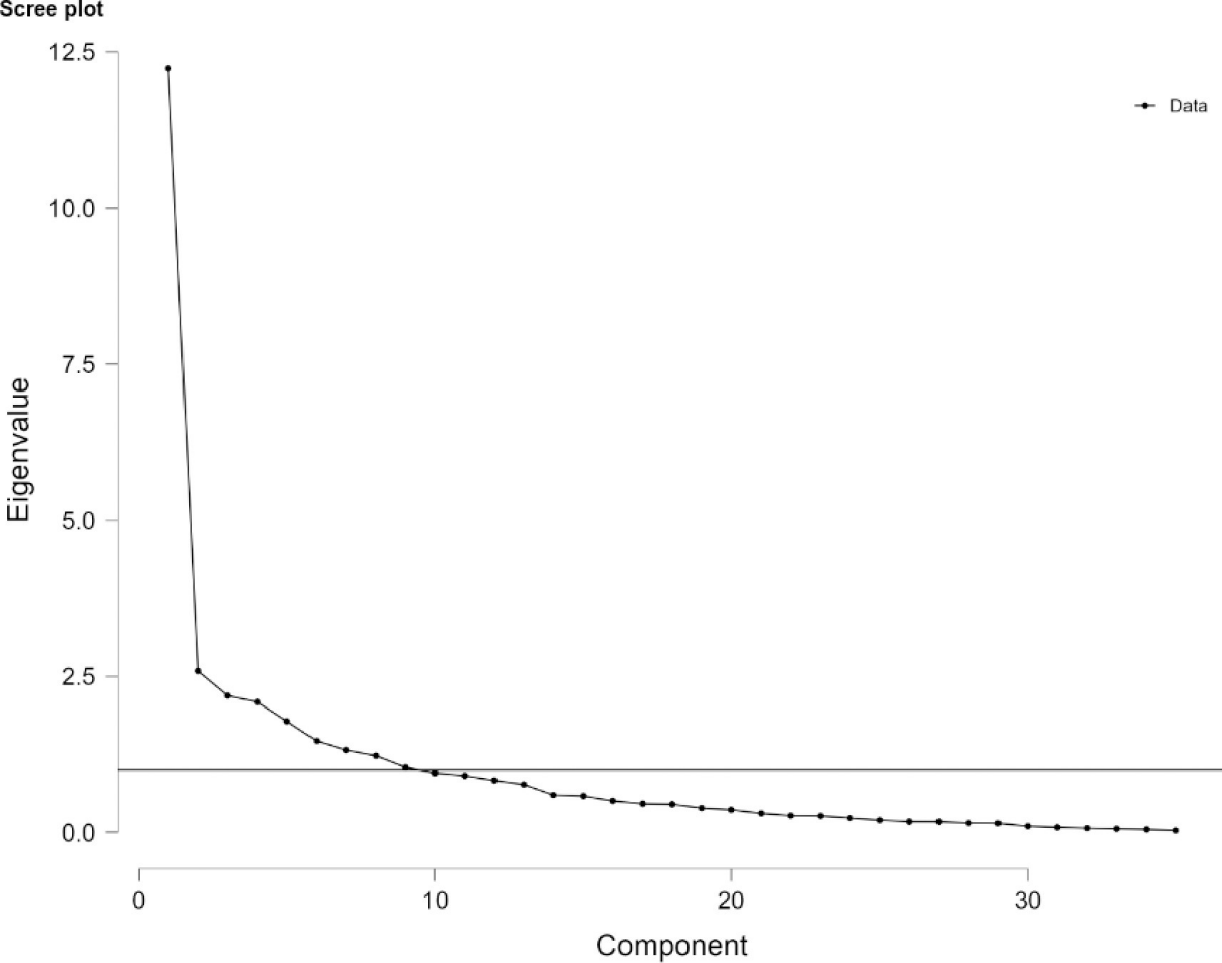
Screeplot.

The “Method Agreement Procedure” [48, 55] highlighted that the choice of 9 dimensions is supported by 3 (21.43%) methods out of 14 (Table 2). To explore more on these dimensions, Principal Component Analysis (PCA) with orthogonal rotation (varimax) was applied and 35 factors were discovered (i.e., as many eigenvectors as the variables). The eigenvalue associated with each factor reflected the variance explained by that factor. Each eigenvalue was also converted into the proportion of variance explained (e.g., factor 1 explained 34.90% of total variance). The first 9 factors explained a cumulative variance (74.10%) above the 70% threshold we set in the methods section (Table 3, Fig 3) and fulfilled the Kaiser’s criterion of eigenvalues>1. Given the aforementioned results nine factors were extracted.

**Table 2:** Results of the “method agreement procedure” [37, 46], regarding how many factors are hidden in the dataset provided.

Number of factors	Method
1	Acceleration factor
1	Scree (R2)
3	CNG
4	beta
9	Optimal coordinates
9	Parallel analysis
9	Kaiser criterion
16	Scree (SE)
20	Bartlett
20	Bentler
28	t
28	p
29	Lawley
30	Anderson

**Table 3:** Exploring the eigenvalues of the 35 items of questionnaire, and extracting the 9 dimensions identified.

Factor	Initial Eigenvalues*			Extraction Sums of Squared Loadings **			Rotation Sums of Squared Loadings **		
	Total	% of Variance	Cumulative %	Total	% of Variance	Cumulative %	Total	% of Variance	Cumulative %
1	12.232	34.90	34.90	11.928	34.1	34.1	3.620	10.3	10.3
2	2.589	7.40	42.30	2.361	6.7	40.8	3.382	9.7	20.0
3	2.193	6.30	48.60	1.845	5.3	46.1	3.356	9.6	29.6
4	2.092	6.00	54.60	1.742	5.0	51.1	2.949	8.4	38.0
5	1.779	5.10	59.70	1.432	4.1	55.2	2.514	7.2	45.2
6	1.464	4.20	63.90	1.144	3.3	58.4	2.137	6.1	51.3
7	1.319	3.80	67.60	0.986	2.8	61.3	2.005	5.7	57.0
8	1.228	3.50	71.10	0.917	2.6	63.9	1.557	4.4	61.5
9	1.041	3.00	74.10	0.671	1.9	65.8	1.531	4.4	65.9
10	0.953	2.70	76.80						
11	0.909	2.60	79.40						
12	0.833	2.40	81.80						
13	0.769	2.20	84.00						
14	0.599	1.70	85.70						
15	0.583	1.70	87.40						
16	0.505	1.40	88.80						
17	0.458	1.30	90.10						
18	0.450	1.30	91.40						
19	0.389	1.10	92.50						
20	0.361	1.00	93.60						
21	0.303	0.90	94.40						
22	0.267	0.80	95.20						
23	0.262	0.70	95.90						
24	0.229	0.70	96.60						
25	0.195	0.60	97.10						
26	0.171	0.50	97.60						
27	0.169	0.50	98.10						
28	0.148	0.40	98.50						
29	0.143	0.40	99.00						
30	0.097	0.30	99.20						
31	0.078	0.20	99.50						
32	0.065	0.20	99.60						
33	0.054	0.20	99.80						
34	0.045	0.10	99.90						
35	0.028	0.08	100.00						

* Estimating the eigenvalues of all the 35 items with Principal Component Analysis (PCA)

** Estimating the extracted, nine factor loadings after applying Exploratory Factor Analysis (EFA) with a weighted least squares (WLS) procedure and a varimax rotation

We continued by applying EFA, with a weighted least squares (WLS) procedure and a varimax rotation, and extracted the nine factors that had eigenvalues>1 (Table 3). Varimax rotation helped us to assess further the factor structure. Factor 1 accounted for more variance (34.1%) than the other eight (6.7%, 5.3%, 5.0%, 4.1%, 3.3%, 2.8%, 2.6% and 1.9%) before rotation. After rotation it accounted for 10.2% of variance (compared to 9.7%, 9.6%, 8.4%, 7.2%, 6.1%, 5.7%, 4.4% and 4.4% of the rest of the factors).

Finally, we evaluated the nine extracted dimensions in relation to the initial, theoretical 12 sub-scales. S4 Table shows how each question loads onto each of the nine extracted factors. The sub-scales that had their items loaded together onto common factors were perceived convenience (i.e., Factor 2), perceived outcome (i.e., Factor 4), perceived medical risk (i.e., Factor 1), perceived information risk (i.e., Factor 5), emotional preference (i.e., Factor 7), perceived medical liability (i.e., Factor 6), subjective norm (i.e., Factor 3), perceived behavioral control (i.e., Factor 9), and behavioral intention (i.e., Factor 1 or Factor 4).

### Reliability

The reliability measurements of all the 35 items was 0.938, 0.979, 0.941 for Cronbach’s α, Guttman’s λ6, and McDonald’s ω respectively. These values indicated very good reliability while the same reliability measurements for each of the 12 domains, with more than two-items, ranged from 0.544 to 0.899 (Shown in Table 4). Perceived medical liability (PML) was the sub-domain with the worst reliability measurements, having 0.603, 0.544, 0.710 for Cronbach’s α, Guttman’s λ6, and McDonald’s ω respectively. The rest of the domains had reliability measurements close to 0.8 (i.e., Perceived Behavioral Control, PBC) and above.

**Table 4:** Reliability analysis for each of the dimensions, with more than two items, of the questionnaire.

Dimension	a*	λ6**	ω***	Average inter-item correlation	Question/item	If item dropped			Item-rest correlation
						a*	λ6**	ω***	
**Perceived convenience, PC**	0.846	0.850	0.848	0.542	PC1	0.824	0.828	0.828	0.619
					PC2	0.810	0.798	0.815	0.682
					PC3	0.789	0.757	0.794	0.766
					PC4	0.791	0.783	0.794	0.734
					PC5	0.857	0.838	0.859	0.525
**Perceived outcome, PO**	0.866	0.822	0.875	0.685	PO1	0.866	0.866	0.866	0.686
					PO2	0.754	0.754	0.754	0.805
					PO3	0.803	0.803	0.803	0.759
**Perceived medical risk, PMR**	0.870	0.884	0.875	0.577	PMR1	0.826	0.843	0.837	0.761
					PMR2	0.828	0.819	0.833	0.751
					PMR3	0.833	0.813	0.836	0.735
					PMR4	0.867	0.867	0.881	0.606
					PMR5	0.857	0.845	0.863	0.645
**Perceived medical liability, PML**	0.603	0.544	0.710	0.330	PML1	0.681	0.518	0.681	0.265
					PML2	0.225	0.132	0.224	0.582
					PML3	0.497	0.338	0.496	0.421
**Attitude toward the behavior. ATTB**	0.868	0.874	0.899	0.612	ATTB1	0.785	0.673	0.785	0.533
					ATTB2	0.631	0.467	0.631	0.679
					ATTB3	0.667	0.504	0.666	0.664
**Subjective norm, SN**	0.868	0.874	0.899	0.612	SN1	0.778	0.785	0.841	0.785
					SN2	0.766	0.757	0.822	0.757
					SN3	0.789	0.794	0.843	0.794
					SN4	0.948	0.928	0.948	0.928
**Perceived behavioral control, PBC**	0.771	0.735	0.822	0.530	PBC1	0.760	0.613	0.760	0.544
					PBC2	0.503	0.344	0.502	0.756
					PBC3	0.764	0.634	0.764	0.545
**Pooled (All 35 items)**	0.938	0.979	0.941						

* α: Cronbach’s α

** λ6: Guttman’s λ6

*** ω: McDonald’s ω

Most of the dimensions with more than two-items had an average inter-item correlation, ranging from 0.530 to 0.685. Perceived medical liability (PML) had a low average inter-item correlation with a value of 0.330. Regarding the separate items in each of these dimensions PC5 and PML1 had a moderate to low correlation (i.e., 0.525 and 0.265, respectively) with the other items in their domain. When they were omitted the overall reliability of their dimension improved (e.g., Cronbach’s α increased from 0.846 to 0.857 for PC5, and 0.603 to 0.681 for PML1). The PML dimension had the lowest item-rest correlations among its items (0.265 for PML1, 0.582 for PML2, 0.421 for PML3).

Regarding the two-item sub-scales of the questionnaire, health consciousness (HC) had a low Spearman-Brown reliability with a value of 0.356. The other three, meaning perceived information risk (PIR), emotional preference (EP) and behavioral intention (BI) had good Spearman-Brown reliability, with a value of 0.753, 0.778 and 0.896 respectively.

## Discussion

The purpose of the current study was the translation and cultural adaptation of a questionnaire to measure telemedicine acceptance into the Greek context. Furthermore, the examination of its psychometric properties. Currently, there is a lack of appropriate tools to assess the acceptance of telemedicine by Greek patients. Nevertheless, the need to implement and deploy telemedicine services, besides to remote geographical areas, has been highlighted significantly during the pandemic [35, 62–64]. This service gap has affected not only COVID-19 patients, but also chronic patients and patients from poor socio-economic background. We hope that the adaptation and on-going validation of this questionnaire will support Greek researchers and telemedicine providers to scrutinize their solutions and adjust them to different patient cohorts, besides citizens of remote areas. We hope, also, that it will inform clinical decision and health policy makers with possible gaps for the integration of telemedicine in the current care flow.

### Strengths and weaknesses

The translation and cultural adaptation of the original questionnaire proceeded with a pilot study to get insights about its construct validity. Our pilot study, identified nine (9) latent dimensions among the twelve (12) initial, theoretical constructs of the questionnaire. This is in accordance with the validation stage of the original questionnaire, where Dehe Li et al., 2020 [36] validated and separated nine (9) domains after the confirmatory factory analysis. Nevertheless, we identified attitude toward the behavior (ATTB), health consciousness (HC) and perceived severity of disease (PSD) to be problematic, while Dehe Li et al., 2020 [36] identified perceived medical liability (PML), perceived outcome (PO) and subjective norms (SN) to be as such. This discrepancy could be due to our sample size (n = 73) or the different characteristics of the two cohorts. Our sample had older participants and, probably, patients with a more burdensome clinical profile. For example, while we had only one (1) (1.4%) participant aged 19–29 years from an overall sample of 73 women with breast cancer, the study from Dehe Li et al., 2020 [36] had 332 participants (52.%) aged 19–29 years from an overall sample of 638 participants with an undefined level of disease severity. Further data collection and analyses will be needed to define, in a more generalised way, how the type of patients and the sample size of participants can affect the dimensions extracted and validated.

The created questionnaire had good overall and internal reliability scores for most of its sub-scales. Nevertheless, perceived medical liability (PML) showed medium reliability measurements (a = 0.603, λ6 = 0.544, ω = 0.710, Table 4) and low average inter-item correlation (0.33, Table 4) while health consciousness (HC) showed low Spearman-Brown reliability (0.356, Table 5). On another note, several items (i.e., PMR1 to PMR5, PIR1 and PIR2, PML1 to PML3, PSD1, HC1) across its sub-scales need rewording to take a positive direction (Fig 2, S1 Fig). Finally, while the analysis did not identify the same number of latent dimensions as the original theoretical model, some of them did not load consistently on the 9 identified ones (S4 Table). For example, the items for attitude toward the behavior (ATTB) and health consciousness (HC) loaded onto separate factors. Also, perceived severity of disease (PSD) had a negative loading plus a negative correlation with several questionnaire items.

**Table 5:** Reliability analysis for each of the two-item dimensions of the questionnaire.

Dimension	Spearman-Brown reliability
Perceived information risk, PIR	0.753
Emotional Preference, EP	0.778
Health consciousness, HC	0.356
Behavioral intention	0.896

## Conclusions and future work

This pilot exhibited satisfactory internal reliability scores for the majority of the sub-scales of our translated and culturally adapted Greek questionnaire. Overall, the results of this study were positive. The initial theoretical model and questionnaire had a certain number of latent dimensions; however, factor analysis did not uncover the same number in our case. Reverse-wording items that need to be recoded and items that could be removed from further iterations of the questionnaire were found and categorized.

Future studies will apply this study’s questionnaire to other diverse subpopulations of patients to explore valuable insights and establish norms for the scale for a broader range of patients. Having a bigger sample of patients, running a test-retest longitudinal study, assessing the construct, convergent and discriminant validity are elements to be further examined in future studies. In spite of the current short-comings of this questionnaire, we expect that it will be adapted from future research for telemedicine acceptance by Greek patients. We also expect that it will support future telemedicine and telecare projects in Greece in evaluating the design and implementation of their digital solutions in a more systematic and patient-centered manner.

## Supporting information

S1 Fig Spearman correlation analysis between all the ordinal, likert items of the translated and culturally adapted questionnaire. Items are plotted and grouped together after a hierarchical clustering of their correlation matrix. The 12 rectangles inside the plot are drawn around the items that have the highest correlation between them. You can see all the items at S2 Table. The 12 sub-scales of the questionnaire are: perceived convenience (PC); perceived outcome (PO); perceived medical risk (PMR); perceived information risk (PIR); emotional preference (EP); perceived medical liability (PML); attitude toward the behavior (ATTB); subjective norm (SN); health consciousness (HN); perceived severity of disease (PSD); perceived behavioral control (PBC); behavioral intention (BI).
(DOCX)

S1 Table Cognitive interview questions. (DOCX)

S2 Table Presentation of the Greek adjusted version, and its English translation, of the questionnaire measuring patient’s intention to use online services provided by hospitals. (DOCX)

S3 Table Univariate analysis of the 35 items of the questionnaire (before reverse coding the reverse worderd items). (DOCX)

S4 Table Factor loading results for the 35 items of the questionnaire. The 12 sub-scales of the questionnaire are: perceived convenience (PC); perceived outcome (PO); perceived medical risk (PMR); perceived information risk (PIR); emotional preference (EP); perceived medical liability (PML); attitude toward the behavior (ATTB); subjective norm (SN); health consciousness (HN); perceived severity of disease (PSD); perceived behavioral control (PBC); behavioral intention (BI).
(DOCX)
