# Supplementary material for: Translation, cultural adaptation and pilot testing of a questionnaire measuring the factors affecting the acceptance of telemedicine by Greek cancer patients
Source: PLoS One. 2023 Feb 2;18(2):e0278758. doi: 10.1371/journal.pone.0278758 (PMC9894466; doi:10.1371/journal.pone.0278758)
Supplement: S1 Fig — Items are plotted and grouped together after a hierarchical clustering of their correlation matrix. The 12 rectangles inside the plot are drawn around the items that have the highest correlation between them. You can see all the items at S2 Table. The 12 sub-scales of the questionnaire are: perceived convenience (PC); perceived outcome (PO); perceived medical risk (PMR); perceived information risk (PIR); emotional preference (EP); perceived medical liability (PML); attitude toward the behavior (ATTB); subjective norm (SN); health consciousness (HN); perceived severity of disease (PSD); perceived behavioral control (PBC); behavioral intention (BI). (DOCX) [file pone.0278758.s001.docx]

**Figure S1.** Spearman correlation analysis between all the ordinal, likert items of the translated and culturally adapted questionnaire. Items are plotted and grouped together after a hierarchical clustering of their correlation matrix. The 12 rectangles inside the plot are drawn around the items that have the highest correlation between them. You can see all the items at Table S2.


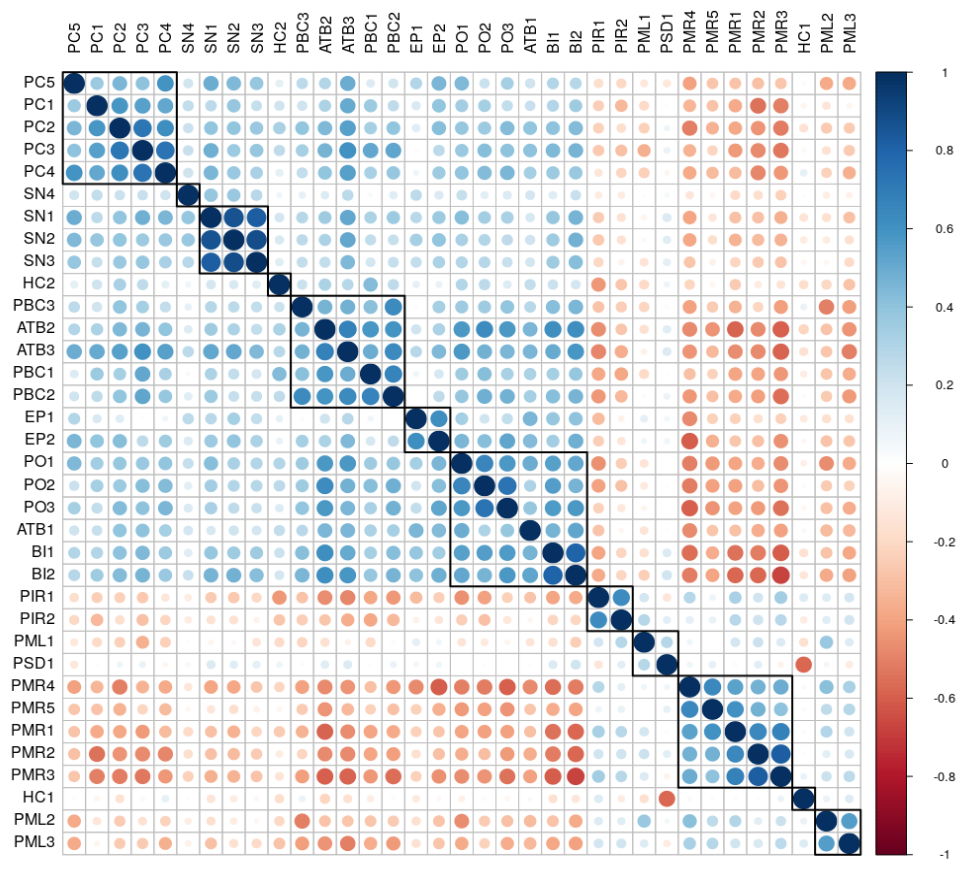


The 12 sub-scales of the questionnaire are: perceived convenience (PC); perceived outcome (PO); perceived medical risk (PMR); perceived information risk (PIR); emotional preference (EP); perceived medical liability (PML); attitude toward the behavior (ATTB); subjective norm (SN); health consciousness (HN); perceived severity of disease (PSD); perceived behavioral control (PBC); behavioral intention (BI)
