# Supplementary material for: Translation, cultural adaptation and pilot testing of a questionnaire measuring the factors affecting the acceptance of telemedicine by Greek cancer patients
Source: PLoS One. 2023 Feb 2;18(2):e0278758. doi: 10.1371/journal.pone.0278758 (PMC9894466; doi:10.1371/journal.pone.0278758)
Supplement: S1 Table — (DOCX) [file pone.0278758.s002.docx]

**Table S1:** Cognitive interview questions

| The following questions are to be made in stages, across the participant’s engagement to the main questionnaire. | |
| --- | --- |
| Οδηγίες συμπλήρωσης ερωτηματολογίου. Θα ερωτηθούν μια φορά σε κάθε συμμετέχοντα, αφού πρώτα διαβάσουν τις οδηγίες συμπλήρωσης του ερωτηματολογίου. | Instructions for completing the questionnaire. They will be asked once to each participant, after first reading the instructions for completing the questionnaire. |
| 1. Με ποιο τρόπο θα κάνατε πιο κατανοητές, τις οδηγίες/κατευθύνσεις για την συμπλήρωση του ερωτηματολογίου; | 1. How would you make the instructions/directions for completing the questionnaire more understandable? |
| 2. Τι σημαίνει για εσάς “μια ηλεκτρονική εφαρμογή παροχής υπηρεσιών υγείας”; | 2. What does an "electronic health service" mean to you? |
| Στοιχεία ερωτηματολογίου. Οι παρακάτω ερωτήσεις -3 έως 8 - θα πρέπει να ερωτηθούν στους συμμετέχοντες της πιλοτικής έρευνας, για κάθε μια ερώτηση του ερωτηματολογίου ξεχωριστά. | Questionnaire's elements. Τhe following questions - 3 to 8 - should be asked to the participant, for each question of the questionnaire separately. |
| 3. Με δικές σας λέξεις, σε τι πιστεύετε ότι αναφέρεται η κάθε ερώτηση/πρόταση; | 3. In your own words, what do you think each question/suggestion refers to? |
| 4. Τι σημαίνει για εσάς η κάθε ερώτηση; Τι σκεφτήκατε όταν απαντούσατε την ερώτηση; | 4. What does each question mean to you? What did you think when you answered the question? |
| 5. Ήταν κατανοητή η ερώτηση; Υπάρχουν κάποιες συγκεκριμένες λέξεις στην ερώτηση/πρόταση που ήταν δύσκολο να τις καταλάβετε; | 5. Was the question understandable? Are there any specific words in the question/sentence that were difficult to understand? |
| 6. Πως θα αλλάζατε τις λέξεις αυτές ώστε να είναι περισσότερο κατανοητές; | 6. How would you change these words to make them more understandable? |
| 7. Ήταν δύσκολο να απαντηθεί η συγκεκριμένη ερώτηση; Εάν ναι, γιατί; | 7. Was this question challenging to answer? If so, why? |
| 8. Πως επιλέξατε ποια απάντησή θα δώσετε ; | 8 .How did you choose which answer to give? |
| Διαστάσεις ερωτηματολογίου. Οι παρακάτω ερωτήσεις -9 έως 11 - θα πρέπει να ερωτηθούν στους συμμετέχοντες της πιλοτικής έρευνας, για κάθε διάσταση του ερωτηματολογίου. Οι διαστάσεις του ερωτηματολογίου είναι Αντιληπτή Άνεση, Αντιληπτά Αποτελέσματα, Αντιληπτός Ιατρικός Κίνδυνος, Αντιληπτός Κίνδυνος Πληροφοριών, Συναισθηματική Προτίμηση, Αντιληπτή Ιατρική Ευθύνη, Στάση – Συμπεριφορά, Υποκειμενικοί Κανόνες, Συνειδητοποίηση για την Κατάσταση της Υγείας, Αντιληπτή Σοβαρότητα της Νόσου, Αντιληπτός Έλεγχος Συμπεριφοράς, Προθεση Συμπεριφοράς | Questionnaire's dimensions. The following questions - 9 to 11 - should be asked to the participant for each dimension of the questionnaire. The dimensions of the questionnaire are Perceived Convience, Perceived Outcome, Perceived Medical Risk, Perceived Information Risk, Emotional Preference, Perceived Medical Liability, Attitude Toward the Behaviour, Subjective Norm, Health consciousness, Perceived Severity of Disease, Perceived Behavioral Control, Behavioral Intention |
| 9. Με δικές σας λέξεις, σε τι πιστεύετε ότι ζητάει η κάθε ομάδα ερωτήσεων; | 9. In your own words, what do you think each question group is asking? |
| 10. Πως πιστεύετε ότι οι ερωτήσεις της κάθε ομάδας, σχετίζονται μεταξύ τους; | 10. How do you think the questions of each group are related to each other? |
| 11. Πιστεύετε ότι κάποια ή κάποιες ερωτήσεις δεν ταιριάζουν σε αυτή την ομάδα; | 11. Do you think any of the questions do not fit into this group? |
| Επιλογές απάντησης. Οι παρακάτω ερωτήσεις -12 έως 13 - θα πρέπει να ερωτηθούν μια φορά σε κάθε συμμετέχοντα, αφού θα έχει ολοκληρώσει τη συμπλήρωση του ερωτηματολογίου. | Answer options. The following questions - 12 to 13 - should be asked once to each participant after completing the questionnaire. |
| 12. Τι πιστεύετε για τις επιλογές που είχατε ως προς την απάντηση των ερωτήσεων. Διαφωνώ απόλυτα, Διαφωνώ, Ουδέτερος, Συμφωνώ, Συμφωνώ Απόλυτα. | 12. What do you think about the choices you had in answering the questions. Strongly Disagree, Disagree, Neutral, Agree, Strongly Agree. |
| 13. Πώς θα κάνατε τις επιλογές απαντήσεων πιο εύκολα κατανοητές; | 13. How would you make the answer choices easier to understand? |
| Συνολική αξιολόγηση. Οι παρακάτω ερωτήσεις -14 έως 16 - θα πρέπει να ερωτηθούν μια φορά σε κάθε συμμετέχοντα, αφού θα έχει ολοκληρώσει τη συμπλήρωση του ερωτηματολογίου. | Total evaluation. The following questions -14 to 16 - should be asked once to each participant after completing the questionnaire. |
| 14. Υπάρχουν ζητήματα τα οποία παραλείψαμε να ρωτήσουμε και είναι κατά τη γνώμη σας σημαντικά; | 14. Are there any issues that we may have failed to ask and are essential to you? |
| 15. Ποιες είναι οι σκέψεις σας/συνολική άποψη για το ερωτηματολόγιο | 15. What are your thoughts/overall opinion on the questionnaire? |
| 16. Υπάρχει κάτι που θα αλλάζατε στο ερωτηματολόγιο συνολικά; | 16. Is there anything you would change about the questionnaire as a whole? |
