# Supplementary material for: Translation, cultural adaptation and pilot testing of a questionnaire measuring the factors affecting the acceptance of telemedicine by Greek cancer patients
Source: PLoS One. 2023 Feb 2;18(2):e0278758. doi: 10.1371/journal.pone.0278758 (PMC9894466; doi:10.1371/journal.pone.0278758)
Supplement: S2 Table — (DOCX) [file pone.0278758.s003.docx]

**Table S2:** Presentation of the Greek adjusted version, and its English Translation, of the questionnaire measuring patient’s intention to use online services provided by hospitals

| Response on a 5-point Likert scale: 1 (Disagree very much) to 5 (Agree very much) | |
| --- | --- |
| Αντιληπτή άνεση | Perceived convenience, PC (sub-scale) |
| 1. Πιστεύω ότι θα ήταν βολικό να χρησιμοποιήσω μια ηλεκτρονική εφαρμογή παροχής υπηρεσιών υγείας ενός νοσοκομείου, από οπουδήποτε, χωρίς να χρειάζεται να μεταβώ σε αυτό, αυτοπροσώπως. | 1. I think it would be convenient to use an electronic health service provided by a hospital anywhere, without going to the hospital in person. |
| 2. Πιστεύω ότι μια ηλεκτρονική εφαρμογή παροχής υπηρεσιών υγείας ενός νοσοκομείου θα μπορούσε να μου παρέχει τις ιατρικές πληροφορίες που χρειάζομαι και να με βοηθήσει να βρω «ευρέως γνωστά» νοσοκομεία ή γιατρούς σε όλη τη χώρα. | 2. I think an electronic health service provided by a hospital could offer a vast amount of medical information and help me find well-known hospitals or doctors across the country. |
| 3. Πιστεύω ότι η χρήση μιας ηλεκτρονικής εφαρμογής παροχής υπηρεσιών υγείας ενός νοσοκομείου θα με βοηθούσε να εξοικονομήσω χρόνο. | 3. I think it would be time-saving to use an electronic health service provided by a hospital. |
| 4. Πιστεύω ότι η χρήση μιας ηλεκτρονικής εφαρμογής παροχής υπηρεσιών υγείας ενός νοσοκομείου θα με βοηθούσε να εξοικονομήσω δαπάνες (π.χ. έξοδα μεταφοράς σε νοσοκομείο, έξοδα διαμονής) | 4. I think that using an electronic health service provided by a hospital could be helpful to save expenses such as transportation and lodging fees. |
| 5. Πιστεύω ότι η χρήση μιας ηλεκτρονικής εφαρμογής παροχής υπηρεσιών υγείας ενός νοσοκομείου θα με βοηθούσε να αποφύγω πιθανές απώλειες, όπως π.χ. η απώλεια εισοδήματος λόγω ασθένειας. | 5. I think that using an electronic health service provided by a hospital could be helpful to decrease losses such as the loss of expected income due to sick leaves. |
| Αντιληπτά αποτελέσματα | Perceived outcome, PO (sub-scale) |
| 1. Πιστεύω ότι μια ηλεκτρονική εφαρμογή παροχής υπηρεσιών υγείας ενός νοσοκομείου θα μπορούσε να κατανοήσει με ακρίβεια την κατάσταση της υγείας μου. | 1. I think that an electronic health service provided by a hospital would be helpful to understand the condition of my health accurately. |
| 2. Πιστεύω ότι μια ηλεκτρονική εφαρμογή παροχής υπηρεσιών υγείας ενός νοσοκομείου θα μπορούσε να μου παρέχει αποτελεσματικές θεραπευτικές συστάσεις. | 2. I think that effective treatment recommendations could be obtained through an electronic health service provided by a hospital. |
| 3. Πιστεύω ότι μια ηλεκτρονική εφαρμογή παροχής υπηρεσιών υγείας ενός νοσοκομείου θα μπορούσε να μου παράσχει αποτελεσματικές οδηγίες για την φαρμακευτική αγωγή. | 3. I think that effective medication guidance could be obtained through an electronic health service provided by a hospital. |
| Αντιληπτός ιατρικός κίνδυνος | Perceived medical risk, PMR (sub-scale) |
| 1. Ανησυχώ επειδή πιστεύω πως αν λάμβανα υπηρεσίες από μια ηλεκτρονική εφαρμογή παροχής υπηρεσιών υγείας ενός νοσοκομείου, οι γιατροί θα έδιναν λιγότερη προσοχή για την κατάσταση της υγείας μου. | 1. I am worried that physicians would pay less attention to my health condition if I used an electronic health service provided by a hospital. |
| 2. Θα είχα αμφιβολίες για τον επαγγελματισμό ενός νοσοκομείου ή των γιατρών του , που παρέχουν υπηρεσίες μέσω μιας ηλεκτρονικής εφαρμογής παροχής υπηρεσιών υγείας ενός νοσοκομείου. | 2. I would have doubts about the professionalism of a hospital or its physicians, which provides an electronic health service. |
| 3. Θα είχα αμφιβολίες για την διαπίστευση των ιατρών και του νοσοκομείου εάν λάμβανα υπηρεσίες από μια ηλεκτρονική εφαρμογή παροχής υπηρεσιών υγείας ενός νοσοκομείου. | 3. I have doubts about the authenticity of a hospital or its physicians, which provides an electronic health service. |
| 4. Ανησυχώ για το ότι ενδέχεται να μην μπορώ να εξηγήσω με σαφήνεια την κατάστασή μου στους γιατρούς, αν χρησιμοποιήσω μια ηλεκτρονική εφαρμογή παροχής υπηρεσιών υγείας ενός νοσοκομείου. | 4. I am worried that I may not clearly explain my conditions to physicians when using an electronic health service provided by a hospital. |
| 5. Εάν λάμβανα υπηρεσίες από μια ηλεκτρονική εφαρμογή παροχής υπηρεσιών υγείας ενός νοσοκομείου, θα ανησυχούσα για πιθανή εσφαλμένη διάγνωση ή ελλιπή διάγνωση, καθώς οι ηλεκτρονικές υπηρεσίες δεν προσφέρουν τη δυνατότητα διεξαγωγής ιατρικών εξετάσεων. | 5. I am worried that because an electronic health service provided by a hospital cannot provide medical examinations, it may result in misdiagnosis and missed diagnosis. |
| Αντιληπτός κίνδυνος πληροφοριών | Perceived information risk, PIR (sub-scale) |
| 1. Ανησυχώ ότι μπορεί να πέσω θύμα απάτης, αν χρησιμοποιήσω μια ηλεκτρονική εφαρμογή παροχής υπηρεσιών υγείας ενός νοσοκομείου. | 1. I am worried that I would be deceived when using an electronic health service provided by a hospital. |
| 2. Ανησυχώ ότι τα προσωπικά στοιχεία θα μπορούσαν να διαρρεύσουν, αν χρησιμοποιήσω μια ηλεκτρονική εφαρμογή παροχής υπηρεσιών υγείας ενός νοσοκομείου. | 2. I am worried that the personal information could be disclosed after using an electronic health service provided by a hospital. |
| Συναισθηματική προτίμηση | Emotional preference, EP (sub-scale) |
| 1. Πιστεύω ότι μέσω της χρήσης μιας ηλεκτρονικής εφαρμογής παροχής υπηρεσιών υγείας ενός νοσοκομείου, θα μπορούσα να αποφύγω την ένταση που υπάρχει στην διαπροσωπική επικοινωνία με τους ιατρούς. | 1. I think using an electronic health service provided by a hospital could avoid the tension associated with face-to-face communication with physicians. |
| 2. Πιστεύω ότι η χρήση μιας ηλεκτρονικής εφαρμογής παροχής υπηρεσιών υγείας ενός νοσοκομείου θα μπορούσε να είναι χρήσιμη για την προστασία των προσωπικών μου και την αποφυγή της αμηχανίας που σχετίζεται με την διαπροσωπική επικοινωνία με τους ιατρούς. | 2. I think that using an electronic health service provided by a hospital could be helpful to protect personal information and avoid the embarrassment associated with face-to-face communication with physicians. |
| Αντιληπτή ιατρική ευθύνη | Perceived medical liability, PML (sub-scale) |
| 1. Πιστεύω ότι οι σχετικοί νόμοι και κανονισμοί που αφορούν την παροχή ηλεκτρονικών υπηρεσιών υγεία από τα νοσοκομεία βρίσκονται σε ημιτελές στάδιο. | 1. I think the relevant laws and regulations about an electronic health service provided by a hospital are at an imperfect stage. |
| 2. Πιστεύω ότι είναι δύσκολο να διαχωριστεί η ευθύνη των ιατρικών λαθών που είναι πιθανό να προκύψουν κατά τη χρήση μιας ηλεκτρονικής εφαρμογής παροχής υπηρεσιών υγείας ενός νοσοκομείου, μεταξύ ασθενών, ιατρών, νοσοκομείων και διαδικτυακών πλατφορμών. | 2. I think it is difficult to divide the liability of medical accidents encountered between patients, physicians, hospitals, and network platforms when using an electronic health service provided by a hospital. |
| 3. Πιστεύω ότι θα ήταν δύσκολο να προστατέψω τα νομικά μου δικαιώματα, εάν συνέβαινε ένα ιατρικό λάθος κατά τη χρήση μιας ηλεκτρονικής εφαρμογής παροχής υπηρεσιών υγείας ενός νοσοκομείου. | 3. I think it would be difficult to protect my legal rights when a medical accident occurs while using the online inquiry services provided by internet hospitals. |
| Στάση - Συμπεριφορά | Attitude toward the behavior. ATTB (sub-scale) |
| 1. Γενικά πιστεύω ότι η εμπειρία της χρήσης μιας ηλεκτρονικής εφαρμογής παροχής υπηρεσιών υγείας ενός νοσοκομείου θα είναι καλύτερη από αυτή των υπηρεσιών που παρέχονται από τα παραδοσιακά νοσοκομεία. | 1. Overall, I think using an electronic health service provided by a hospital will be a better experience than those offered by traditional hospitals. |
| 2. Γενικά πιστεύω ότι μια ηλεκτρονική εφαρμογή παροχής υπηρεσιών υγείας ενός νοσοκομείου είναι χρήσιμη για τη θεραπεία ασθενειών. | 2. Overall, I think using an electronic health service provided by a hospital is helpful for disease treatment. |
| 3. Γενικά πιστεύω ότι έχει νόημα η χρήση μιας ηλεκτρονικής εφαρμογής παροχής υπηρεσιών υγείας ενός νοσοκομείου. | 3. Overall, I think it is meaningful to use an electronic health service provided by a hospital. |
| Υποκειμενικοί κανόνες | Subjective norm, SN (sub-scale) |
| 1. Η οικογένεια όσο και οι φίλοι μου πιστεύουν ότι πρέπει να χρησιμοποιήσω ηλεκτρονικές υπηρεσίες υγείας ενός νοσοκομείου. | 1. Both family and friends around me think I should use an electronic health service provided by a hospital. |
| 2. Άλλοι ασθενείς από τον κοινωνικό μου περίγυρο, πιστεύουν ότι πρέπει να χρησιμοποιήσω ηλεκτρονικές υπηρεσίες υγείας ενός νοσοκομείου. | 2. Patients around me think I should use an electronic health service provided by a hospital. |
| 3. Οι «φίλοι» μου στο διαδίκτυο πιστεύουν ότι πρέπει να χρησιμοποιήσω ηλεκτρονικές υπηρεσίες υγείας ενός νοσοκομείου. | 3. Web friends think I should use an electronic health service provided by a hospital. |
| 4. Η κυβέρνηση υποστηρίζει τη χρήση ηλεκτρονικών υπηρεσιών υγείας ενός νοσοκομείου. | 4. The government has been advocating using an electronic health service provided by a hospital. |
| Συνειδητοποίηση για την κατάσταση της υγείας | Health consciousness, HC (sub-scale) |
| 1. Είμαι ενήμερος για τα προβλήματα υγείας μου και ανησυχώ πολύ γι’ αυτά. | 1. I am aware of and very concerned about my health problems. |
| 2. Θα προσπαθήσω να διαχειριστώ και να βελτιώσω το επίπεδο ευεξίας μου. | 2. I will try to manage and improve my wellness. |
| Αντιληπτή σοβαρότητα νόσου | Perceived severity of disease, PSOD (sub-scale) |
| 1. Δεν πιστεύω ότι τα προβλήματα υγείας μου είναι σοβαρά. | 1. I do not think my health problems are serious. |
| Αντιληπτός έλεγχος συμπεριφοράς | Perceived behavioral control, PBC (sub-scale) |
| 1. Διαθέτω τον κατάλληλο υλικοτεχνικό εξοπλισμό (π.χ. smartphone, υπολογιστής, σύνδεση με το διαδίκτυο) για να χρησιμοποιήσω την ηλεκτρονική εφαρμογή παροχής υπηρεσιών υγείας ενός νοσοκομείου. | 1. I have appropriate hardware equipment (such as smartphone and computer) to use an electronic health service provided by a hospital. |
| 2. Θα μπορούσα να διαχειριστώ από μόνος μου μια ηλεκτρονική εφαρμογή παροχής υπηρεσιών υγείας ενός νοσοκομείου. | 2. I could independently complete the operation process of using an electronic health service provided by a hospital. |
| 3. Θα μπορούσα με σαφήνεια να διακρίνω τις έγκυρες και μη έγκυρες πληροφορίες σε μια ηλεκτρονική εφαρμογή παροχής υπηρεσιών υγείας ενός νοσοκομείου. | 3. I could clearly distinguish the valid and invalid information about an electronic health service provided by a hospital. |
| Πρόθεση Συμπεριφοράς | Behavioral intention, BI (sub-scale) |
| 1. Αν νιώσω άρρωστος θα επιλέξω να λάβω υπηρεσίες υγείας από την ηλεκτρονική εφαρμογή παροχής υπηρεσιών υγείας ενός νοσοκομείου. | 1. If I feel sick, I will choose to use an electronic health service provided by a hospital. |
| 2. Εάν κάποιος από τον περίγυρό μου χρειαστεί υπηρεσίες υγείας, θα του πρότεινα την χρήση της ηλεκτρονικής εφαρμογής παροχής υπηρεσιών υγείας ενός νοσοκομείου. | 2. If someone around me requires health services, I would recommend using an electronic health service provided by a hospital. |
