# Supplementary material for: Translation, cultural adaptation and pilot testing of a questionnaire measuring the factors affecting the acceptance of telemedicine by Greek cancer patients
Source: PLoS One. 2023 Feb 2;18(2):e0278758. doi: 10.1371/journal.pone.0278758 (PMC9894466; doi:10.1371/journal.pone.0278758)
Supplement: S3 Table — (DOCX) [file pone.0278758.s004.docx]

**Table S3:** Univariate analysis of the 35 items of the questionnaire (before reverse coding the reverse worderd items)

| **Dimension** | **Question** | **Mean** | **SD** | **N** |
| --- | --- | --- | --- | --- |
| **Perceived convenience, PC** | PC1. I think it would be convenient to use an electronic health service provided by a hospital anywhere, without going to the hospital in person. | 4.233 | 0.825 | 73 |
|  | PC2. I think an electronic health service provided by a hospital could offer a vast amount of medical information and help me find well-known hospitals or doctors across the country. | 4.192 | 0.700 | 73 |
|  | PC3. I think it would be time-saving to use an electronic health service provided by a hospital. | 4.274 | 0.712 | 73 |
|  | PC4. I think that using an electronic health service provided by a hospital could be helpful to save expenses such as transportation and lodging fees. | 4.082 | 0.846 | 73 |
|  | PC5. I think that using an electronic health service provided by a hospital could be helpful to decrease losses such as the loss of expected income due to sick leaves. | 3.575 | 0.942 | 73 |
| **Perceived outcome, PO** | PO1. I think that an electronic health service provided by a hospital would be helpful to understand the condition of my health accurately. | 2.603 | 0.862 | 73 |
|  | PO2. I think that effective treatment recommendations could be obtained through an electronic health service provided by a hospital. | 2.904 | 0.960 | 73 |
|  | PO3. I think that effective medication guidance could be obtained through an electronic health service provided by a hospital. | 3.219 | 1.031 | 73 |
| **Perceived medical risk, PMR** | PMR1. I am worried that physicians would pay less attention to my health condition if I used an electronic health service provided by a hospital. | 2.904 | 0.945 | 73 |
|  | PMR2. I would have doubts about the professionalism of a hospital or its physicians, which provides an electronic health service. | 3.233 | 0.965 | 73 |
|  | PMR3. I have doubts about the authenticity of a hospital or its physicians, which provides an electronic health service. | 3.151 | 0.908 | 73 |
|  | PMR4. I am worried that I may not clearly explain my conditions to physicians when using an electronic health service provided by a hospital. | 2.575 | 0.985 | 73 |
|  | PMR5. I am worried that because an electronic health service provided by a hospital cannot provide medical examinations, it may result in misdiagnosis and missed diagnosis. | 2.329 | 0.746 | 73 |
| **Perceived information risk, PIR** | PIR1. I am worried that I would be deceived when using an electronic health service provided by a hospital. | 3.493 | 0.835 | 73 |
|  | PIR2. I am worried that the personal information could be disclosed after using an electronic health service provided by a hospital. | 3.452 | 0.929 | 73 |
| **Emotional preference, EP** | EP1. I think using an electronic health service provided by a hospital could avoid the tension associated with face-to-face communication with physicians. | 2.603 | 0.939 | 73 |
|  | EP2. I think that using an electronic health service provided by a hospital could be helpful to protect personal information and avoid the embarrassment associated with face-to-face communication with physicians. | 2.603 | 0.893 | 73 |
| **Perceived medical liability, PML** | PML1. I think the relevant laws and regulations about an electronic health service provided by a hospital are at an imperfect stage. | 2.205 | 0.745 | 73 |
|  | PML2. I think it is difficult to divide the liability of medical accidents encountered between patients, physicians, hospitals, and network platforms when using an electronic health service provided by a hospital. | 2.288 | 0.92 | 73 |
|  | PML3. I think it would be difficult to protect my legal rights when a medical accident occurs while using the online inquiry services provided by internet hospitals. | 2.795 | 0.999 | 73 |
| **Attitude toward the behavior, ATTB** | ATTB1. Overall, I think using an electronic health service provided by a hospital will be a better experience than those offered by traditional hospitals. | 2.781 | 0.901 | 73 |
|  | ATTB2. Overall, I think using an electronic health service provided by a hospital is helpful for disease treatment. | 3.164 | 1.028 | 73 |
|  | ATTB3. Overall, I think it is meaningful to use an electronic health service provided by a hospital. | 3.712 | 0.772 | 73 |
| **Subjective norm, SN** | SN1. Both family and friends around me think I should use an electronic health service provided by a hospital. | 2.849 | 0.758 | 73 |
|  | SN2. Patients around me think I should use an electronic health service provided by a hospital. | 2.877 | 0.763 | 73 |
|  | SN3. Web friends think I should use an electronic health service provided by a hospital. | 2.795 | 0.816 | 73 |
|  | SN4. The government has been advocating using an electronic health service provided by a hospital. | 3.041 | 0.696 | 73 |
| **Health consciousness, HC** | HC1. I am aware of and very concerned about my health problems. | 2.384 | 0.907 | 73 |
|  | HC2. I will try to manage and improve my wellness. | 4.178 | 0.561 | 73 |
| **Perceived severity of disease, PSD** | PSD1. I do not think my health problems are serious. | 3.397 | 1.024 | 73 |
| **Perceived behavioral control, PBC** | PBC1. I have appropriate hardware equipment (such as smartphone and computer) to use an electronic health service provided by a hospital. | 4.288 | 4.288 | 73 |
|  | PBC2. I could independently complete the operation process of using an electronic health service provided by a hospital. | 3.959 | 0.92 | 73 |
|  | PBC3. I could clearly distinguish the valid and invalid information about an electronic health service provided by a hospital. | 3.151 | 0.923 | 73 |
| **Behavioral intention, BI** | BI1. If I feel sick, I will choose to use an electronic health service provided by a hospital. | 2.685 | 1.039 | 73 |
|  | BI2. If someone around me requires health services, I would recommend using an electronic health service provided by a hospital. | 2.849 | 0.981 | 73 |
