# Supplementary material for: Translation, cultural adaptation and pilot testing of a questionnaire measuring the factors affecting the acceptance of telemedicine by Greek cancer patients
Source: PLoS One. 2023 Feb 2;18(2):e0278758. doi: 10.1371/journal.pone.0278758 (PMC9894466; doi:10.1371/journal.pone.0278758)
Supplement: S4 Table — The 12 sub-scales of the questionnaire are: perceived convenience (PC); perceived outcome (PO); perceived medical risk (PMR); perceived information risk (PIR); emotional preference (EP); perceived medical liability (PML); attitude toward the behavior (ATTB); subjective norm (SN); health consciousness (HN); perceived severity of disease (PSD); perceived behavioral control (PBC); behavioral intention (BI). (DOCX) [file pone.0278758.s005.docx]

**Table S4:** Factor loading results for the 35 items of the questionnaire

| **Item** | **Factor**  **1** | **Factor**  **2** | **Factor 3** | **Factor 4** | **Factor 5** | **Factor 6** | **Factor**  **7** | **Factor**  **8** | **Factor**  **9** | **Communality*** |
| --- | --- | --- | --- | --- | --- | --- | --- | --- | --- | --- |
| PC1 | 0.345 | 0.621 |  |  |  |  |  |  |  | 0.600 |
| PC2 |  | 0.613 |  |  |  |  |  |  |  | 0.578 |
| PC3 |  | 0.795 |  |  |  |  |  |  |  | 0.843 |
| PC4 |  | 0.713 |  |  |  |  |  |  |  | 0.663 |
| PC5 |  | 0.479 |  |  |  | 0.369 | 0.365 |  |  | 0.615 |
| PO1 |  |  |  | 0.480 |  |  | 0.328 |  |  | 0.625 |
| PO2 |  |  |  | 0.745 |  |  |  |  |  | 0.773 |
| PO3 |  |  |  | 0.718 |  |  |  |  |  | 0.762 |
| PMR1 | 0.726 |  |  |  |  |  |  |  |  | 0.668 |
| PMR2 | 0.853 | 0.384 |  |  |  |  |  |  |  | 0.917 |
| PMR3 | 0.715 | 0.326 |  |  |  |  |  |  |  | 0.78 |
| PMR4 | 0.361 |  |  | 0.400 |  |  | 0.463 |  |  | 0.648 |
| PMR5 | 0.513 |  |  |  |  |  | 0.311 |  |  | 0.504 |
| PIR1 |  |  |  |  | 0.808 |  |  |  |  | 0.767 |
| PIR2 |  |  |  |  | 0.727 |  |  |  |  | 0.611 |
| EP1 |  |  |  |  |  |  | 0.696 |  |  | 0.677 |
| EP2 |  |  |  |  |  |  | 0.663 |  |  | 0.671 |
| PML1 |  |  |  |  |  | 0.336 | -0.347 |  |  | 0.536 |
| PML2 |  |  |  |  |  | 0.783 |  |  |  | 0.67 |
| PML3 |  |  |  |  |  | 0.565 |  |  |  | 0.484 |
| ATB1 |  |  |  | 0.314 |  |  |  |  |  | 0.391 |
| ATB2 | 0.404 |  |  | 0.427 |  |  |  |  |  | 0.683 |
| ATB3 |  | 0.497 |  |  |  |  |  |  | 0.336 | 0.711 |
| SN1 |  |  | 0.880 |  |  |  |  |  |  | 0.901 |
| SN2 |  |  | 0.906 |  |  |  |  |  |  | 0.931 |
| SN3 |  |  | 0.926 |  |  |  |  |  |  | 0.893 |
| SN4 |  |  | 0.367 |  |  |  |  |  |  | 0.179 |
| HC1 |  |  |  |  |  |  |  | 0.767 |  | 0.619 |
| HC2 |  |  |  |  | 0.492 |  |  |  |  | 0.327 |
| PSD1 |  |  |  |  |  |  |  | -0.738 |  | 0.569 |
| PBC1 |  |  |  |  | 0.490 |  |  |  | 0.355 | 0.502 |
| PBC2 |  |  |  |  | 0.326 |  |  |  | 0.780 | 0.915 |
| PBC3 |  |  |  |  |  | 0.518 |  |  | 0.474 | 0.654 |
| BI1 | 0.444 |  |  | 0.524 |  |  |  |  |  | 0.664 |
| BI2 | 0.529 |  |  | 0.446 |  |  |  |  |  | 0.721 |
| * Communality: how much an item’s variance is being captured by the factor model | | | | | | | | | | |

The 12 sub-scales of the questionnaire are: perceived convenience (PC); perceived outcome (PO); perceived medical risk (PMR); perceived information risk (PIR); emotional preference (EP); perceived medical liability (PML); attitude toward the behavior (ATTB); subjective norm (SN); health consciousness (HN); perceived severity of disease (PSD); perceived behavioral control (PBC); behavioral intention (BI)
